# *Pseudomonas aeruginosa*-Derived Volatile Sulfur Compounds Promote Distal *Aspergillus fumigatus* Growth and a Synergistic Pathogen-Pathogen Interaction That Increases Pathogenicity in Co-infection

**DOI:** 10.3389/fmicb.2019.02311

**Published:** 2019-10-09

**Authors:** Jennifer Scott, Monica Sueiro-Olivares, Waqar Ahmed, Christoph Heddergott, Can Zhao, Riba Thomas, Michael Bromley, Jean-Paul Latgé, Sven Krappmann, Stephen Fowler, Elaine Bignell, Jorge Amich

**Affiliations:** ^1^Manchester Fungal Infection Group, Division of Infection, Immunity and Respiratory Medicine, Faculty of Biology, Medicine and Health, School of Biological Sciences, University of Manchester, Manchester Academic Health Science Centre, Manchester, United Kingdom; ^2^Respiratory and Allergy Research Group, Division of Infection, Immunity and Respiratory Medicine, Faculty of Biology, Medicine and Health, School of Biological Sciences, University of Manchester, Manchester, United Kingdom; ^3^Manchester Institute of Biotechnology, University of Manchester, Manchester, United Kingdom; ^4^Unité des Aspergillus, Institut Pasteur, Paris, France; ^5^Mikrobiologisches Institut – Klinische Mikrobiologie, Immunologie und Hygiene, Universitätsklinikum Erlangen, Friedrich-Alexander-Universität Erlangen-Nürnberg, Erlangen, Germany; ^6^NIHR Manchester Biomedical Research Centre – Manchester Academic Health Science Centre, Manchester University Hospitals NHS Foundation Trust, Manchester, United Kingdom

**Keywords:** *P. aeruginosa* – *A. fumigatus* interaction, co-infection, volatile sulfur compounds, volatile interaction, interkingdom interaction, polymicrobial infection

## Abstract

Pathogen-pathogen interactions in polymicrobial infections are known to directly impact, often to worsen, disease outcomes. For example, co-infection with *Pseudomonas aeruginosa* and *Aspergillus fumigatus*, respectively the most common bacterial and fungal pathogens isolated from cystic fibrosis (CF) airways, leads to a worsened prognosis. Recent studies of *in vitro* microbial cross-talk demonstrated that *P. aeruginosa*-derived volatile sulfur compounds (VSCs) can promote *A. fumigatus* growth *in vitro*. However, the mechanistic basis of such cross-talk and its physiological relevance during co-infection remains unknown. In this study we combine genetic approaches and GC-MS-mediated volatile analysis to show that *A. fumigatus* assimilates VSCs via cysteine (CysB)- or homocysteine (CysD)-synthase. This process is essential for utilization of VSCs as sulfur sources, since *P. aeruginosa*-derived VSCs trigger growth of *A. fumigatus* wild-type, but not of a Δ*cysB*Δ*cysD* mutant, on sulfur-limiting media. *P. aeruginosa* produces VSCs when infecting *Galleria mellonella* and co-infection with *A. fumigatus* in this model results in a synergistic increase in mortality and of fungal and bacterial burdens. Interestingly, the increment in mortality is much greater with the *A. fumigatus* wild-type than with the Δ*cysB*Δ*cysD* mutant. Therefore, *A. fumigatus’* ability to assimilate *P. aeruginosa* derived VSCs significantly triggers a synergistic association that increases the pathobiology of infection. Finally, we show that *P. aeruginosa* can promote fungal growth when growing on substrates that resemble the lung environment, which suggests that this volatile based synergism is likely to occur during co-infection of the human respiratory airways.

## Introduction

The filamentous fungus *Aspergillus fumigatus* and the bacterium *Pseudomonas aeruginosa* are common respiratory pathogens. Both are able to infect the lungs of immunosuppressed individuals and also of patients with certain underlying diseases, such as bronchiectasis, chronic obstructive pulmonary disease, hospital acquired pneumonia and, particularly, cystic fibrosis (CF). CF is the result of mutations in the CF transmembrane conductance regulator (CFTR) gene, which encodes a chloride channel. Dysfunction of this channel leads to a failure to secrete chloride ions and to increased water absorption from the airway epithelium, resulting in the formation of viscous mucus that is resistant to mucociliary clearance. As a result, a perfect environment for polymicrobial colonization is developed (Ratjen and Doring, 2003).

*Aspergillus fumigatus* and *Pseudomonas aeruginosa* are, respectively, the most prevalent fungal and bacterial pathogens isolated from CF airways (Shoseyov et al., 2006; Amin et al., 2010; Brandt et al., 2018). Recent studies have reported prevalences in CF patients ranging from 31 to 46% for *P. aeruginosa* (Registry CFFP, 2016, Registry UCF, 2016; Reece et al., 2017), up to 57% for *A. fumigatus* (Bakare et al., 2003; Baxter et al., 2013a; Burgel et al., 2016), and a prevalence of 15.8% for co-infection (Zhao et al., 2018). Co-infection with both pathogens is associated with a more rapid decline in CF pulmonary function and more respiratory exacerbations, all leading to a worse prognosis (Amin et al., 2010; Reece et al., 2017). Therefore, both pathogens should be considered and targeted in order to improve CF patient outcomes. In support of this, it was shown that an intravenous antibiotic treatment targeting *Pseudomonas* could also reduce the presence of *Aspergillus* in CF patient sputum. Treatment translated into a greater improvement in the forced expiratory volume in the first second (FEV_1_) for patients initially co-infected than in those only infected with *Pseudomonas* (Baxter et al., 2013b). To understand such cross effects and exploit them to the patients’ benefit a profound insight of the pathogen-pathogen interaction taking place during infection is required.

In the environment microbes constantly interact through molecular signals, which can trigger synergistic and antagonistic associations. Such interactions also occur during polymicrobial infections, and are being increasingly recognized as important pathogenicity factors (Singer, 2010; Peters et al., 2012). The relevance of volatile organic compounds (VOCs), molecular messengers that can exert long distance interactions (Schulz-Bohm et al., 2017), has thus far been largely overlooked. However, the fact that exhaled VOCs are promising diagnostic biomarkers for a number of infections (Ahmed et al., 2017) demonstrates that pathogenic microbes produce these molecules in human tissues, which suggests that they may be at sufficient levels to mediate microbial cross-talk during polymicrobial infections.

Recently, it has been reported that *P. aeruginosa*-derived dimethyl sulfide (DMS) enhances *A. fumigatus* growth *in vitro*, which was proposed to be due to its exploitation as a sulfur source (Briard et al., 2016). We had previously shown that *A. fumigatus* can utilize volatile sulfur compounds (VSCs) as the sole sulfur source (Amich et al., 2013). Therefore, in this study we aimed to unravel the mechanistic basis of *A. fumigatus* assimilation of VSCs, and the relevance of *P. aeruginosa*-derived VSCs for pathogen-pathogen interactions *in vivo*.

## Results

### *A. fumigatus* Assimilates VSCs via Cysteine Synthase-(CysB) and Homocysteine Synthase (CysD) Incorporation of Sulfide

We previously demonstrated that VSCs are produced from the catabolism of methionine (but not from other sulfur sources, Supplementary Figure S1A) and can be assimilated and exploited as the sole sulfur source (Amich et al., 2013). However, it remains unknown whether sulfur acquisition via VSCs is reliant upon gaseous or soluble sources. Aiming to discern between VSC uptake from media or air *A. fumigatus* was cultured on methionine-containing media, in the inner of two concentrically positioned agar plates, for 3 days to promote VSC absorption into sterile S-depleted distal media. At day 3, the inner plate was removed and the outer S-depleted medium ventilated to ensure elimination of any volatiles, after which *A. fumigatus* was inoculated. In this experimental set-up, fungal growth should only occur if the VSCs had been absorbed in the agar media. However, no fungal growth was observed (Supplementary Figure S1B), which suggests that *A. fumigatus* likely assimilates VSCs directly from the air.

**FIGURE 1:**
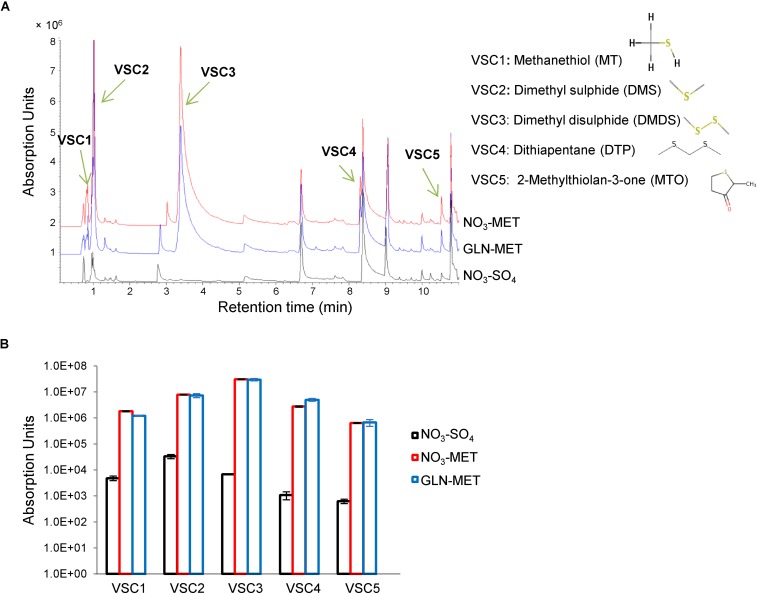
Gas chromatography mass spectrometry (GC-MS) identifies 5 major VSCs produced from *Aspergillus fumigatus* catabolism of methionine. *A. fumigatus* was cultured for 3 days on various solid media before the headspace was analyzed using solid-phase microextraction (SPME) and gas chromatography mass spectrometry (GC-MS) to detect VSCs. **(A)** Chromatogram showing the peaks for the 5 VSCs. These volatiles are produced only on methionine (not on sulfate) and independently of the nitrogen source (ammonium or glutamine). The exact VSCs could be identified and their names and formula are shown. **(B)** Quantification of Absorption Units for each of the volatiles in three biological replicates.

**FIGURE 2:**
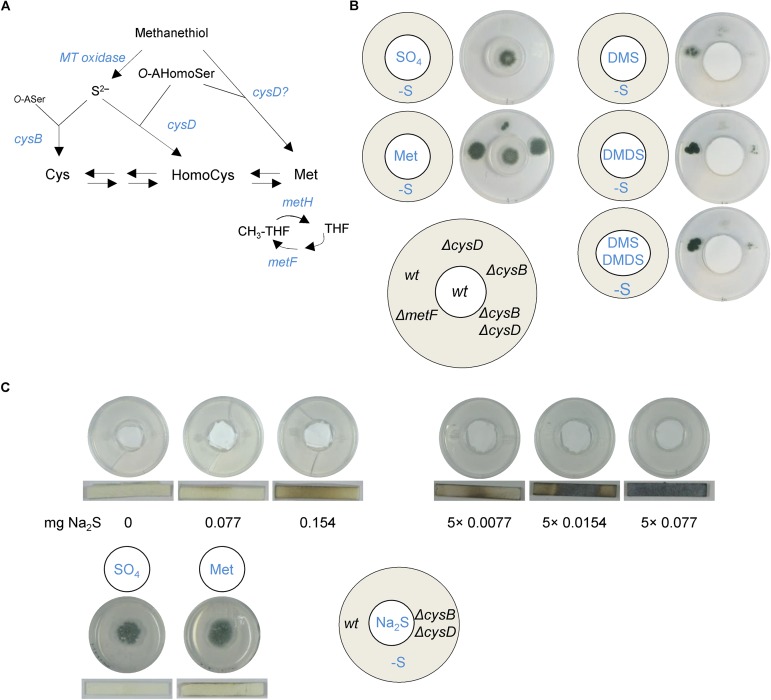
VSCs are incorporated into the trans-sulfuration pathway by the action of CysB and CysD. **(A)** Outline of the possible routes for VSCs assimilation. Sulfide could be liberated from methanethiol (MT) by an MT oxidase action and incorporated into the metabolism through the action of CysB or CysD. Alternatively, MT could be directly incorporated into homocysteine by CysD to yield methionine, bypassing the requirement of MetH (cysB, cysteine synthase; cysD, homocysteine synthase; metH, methionine synthase; metF, methyl-tetrahydrofolate reductase). **(B)** *Aspergillus* derived VSCs and pure DMDS and DMS were not able to trigger growth of a Δ*metF* strain. The volatiles triggered growth of the single Δ*cysB* and Δ*cysD* mutant strains, although to a much lesser extent than the wild-type, but did not trigger growth of the double Δ*cysB*Δ*cysD* mutant. ∼8.5 μg of DMS and ∼10.5 μg of DMDS were added every 12 h (5×) to the filter disk in the inner plate. **(C)** The use of Hydrogen Sulfide Test Strips, which turn dark upon contact with H_2_S, detected that *A. fumigatus* produces low amounts of sulfide (comparable to that liberated from ∼0.077 mg of Na_2_S) from methionine catabolism. Sulfide released from the Na_2_S donor could not trigger growth of the distal *A. fumigatus* colony, even if added in higher amounts or in repeated occasions (5 times, once every 12 h). All plates in **(B,C)** were incubated at 37°C for 3 days.

To precisely identify the VSCs that serve as S-sources, we grew the fungus on minimal medium containing sulfate or methionine as the sole sulfur source and sampled in the headspace of petri dishes using solid-phase microextraction (SPME) and gas chromatography mass spectrometry (GC-MS) (Labows et al., 1980). No VSCs were detected when measuring headspace samples from sterile media. Five major VSCs were present in headspaces sampled from methionine- but not sulfate-cultured *A. fumigatus* (Figure 1) and their production occurred independently of nitrogen metabolism (Figure 1). VSC2 (dimethyl sulfide, DMS) and VSC3 (dimethyl disulfide, DMDS) were previously described to be exploited as S-sources when produced by *P. aeruginosa* (Briard et al., 2016). VSC4 (dithiapentane, DTP) is likely degraded to DMDS (Bellesia et al., 1996), and thus its assimilation likely follows the same route. VSC5 (2-methylthiolan-3-one, MTO) is possibly derived from homocysteine catabolism (Nawrath et al., 2010), but since growth on homocysteine does not trigger distal growth on S-free medium (Supplementary Figure S1A), it is unlikely to be utilized as a major volatile S-source. DMS and DMDS can both be degraded to methanethiol (VSC1), either by a DMDS reductase (Smith and Kelly, 1988), or a DMS monooxygenase (De Bont et al., 1981). Sulfur from methanethiol (MT) could then be assimilated into the metabolism through two different routes. It may be degraded to formaldehyde, hydrogen peroxide, and sulfide by the action of MT oxidase (Suylen et al., 1987; Gould and Kanagawa, 1992), and therefore sulfide (S^2–^) would be incorporated into cysteine by the action of CysB or to homocysteine by the action of CysD (Figure 2A). As an alternative route, MT has also been described to be entirely added to *O*-acetylhomoserine by the action of CysD to directly yield methionine, bypassing the methionine synthase dependent methylation of homocysteine (Bolten et al., 2010; Figure 2A).

To elucidate the assimilation route, we constructed mutant strains deleted in genes that are required for each pathway and investigated their ability to grow on S-free media when exposed to VSCs derived from methionine catabolism or to pure DMDS and DMS (Figure 2B). If CysD was the only gene product responsible for MT assimilation, a Δ*cysD* mutant should not be able to grow on S-free media; however, this strain showed some distal growth (Figure 2B), proving that CysB can also incorporate volatiles and therefore that the MT oxidase route is active. Given that we could not identify any obvious orthologs of MT oxidases described in other microorganisms in *A. fumigatus’* genome, we evaluated the MT oxidase activity potential of *A. fumigatus* protein extracts by measuring the accumulation of formaldehyde upon addition of MT and observed that indeed the fungus seems to have MT oxidase activity (Supplementary Figure S2). To determine if the alternative route is also active we constructed a Δ*metF* strain (Figure 2A) to induce a genetic blockade upon the recycling of methylated tetrahydrofolate (CH_3_-THF), which is required for methionine synthase-dependent methylation of homocysteine. We noticed that Δ*metF* grew very poorly on MM supplemented with methionine, but did grow normally if amino acids (aac) were supplemented (Supplementary Figure S3A); this is not surprising as tetrahydrofolate (THF) mediates the interconversion of serine and glycine and folates play a role in histidine and aromatic amino acid metabolism (Lucock, 2000; Tzin and Galili, 2010). We observed that the Δ*metF* strain was not able to grow on volatiles, implying that the CysD enzyme cannot bypass the MetF dependent requirement of CH_3_-THF to synthesize methionine and therefore that the alternative route is not active (Figure 2B). Finally, a Δ*cysB*Δ*cysD* double mutant could not grow on *A. fumigatus* derived or externally added volatiles, whereas the single mutant strains Δ*cysB* and Δ*cysD* did grow, although to a much lower degree (Figure 2B). This indicates that assimilation of VSCs must go through the conversion of MT into sulfide and its subsequent incorporation by CysD or CysB, and that both enzymatic actions are required for efficient assimilation of S^2–^. Reintroduction of the CysB encoding gene in the Δ*cysB*Δ*cysD* strain reconstituted its ability to grow on sulfate and to cross-feed from VSCs, demonstrating that the growth defect of the double mutant is specific to the mutations (Supplementary Figure S3B).

Therefore, *A. fumigatus* can use DMS and DMDS as the sole S-sources by converting them to sulfide, which is assimilated into the metabolism. This raises the question of whether H_2_S may be solely responsible for the cross-feeding effect. Since our instruments cannot detect H_2_S we used hydrogen sulfide test strips to determine if this gas is produced by *A. fumigatus* and a chemical donor (Na_2_S) to investigate its potential to cross-feed a distal colony. We found that *A. fumigatus* does produce low amounts of H_2_S when grown on methionine, but not on sulfate (Figure 2C). However, a distal *A. fumigatus* colony could not cross-feed from pure sulfide, even if we attempted higher amounts of the compound and also adding it on repeated occasions (Figure 2C). We could differentiate between the absence of cross-feeding effects and the toxicity of H_2_S as the addition of the highest concentrations used (5 × 0.0154 mg and 5 × 0.077 mg) caused the disappearance of the background residual growth (Figure 2C).

### *P. aeruginosa* Derived VSCs Are Exploited as S-Sources by *A. fumigatus*, Promoting Its Growth on Low Concentrations of Organic Sulfur

**FIGURE 3:**
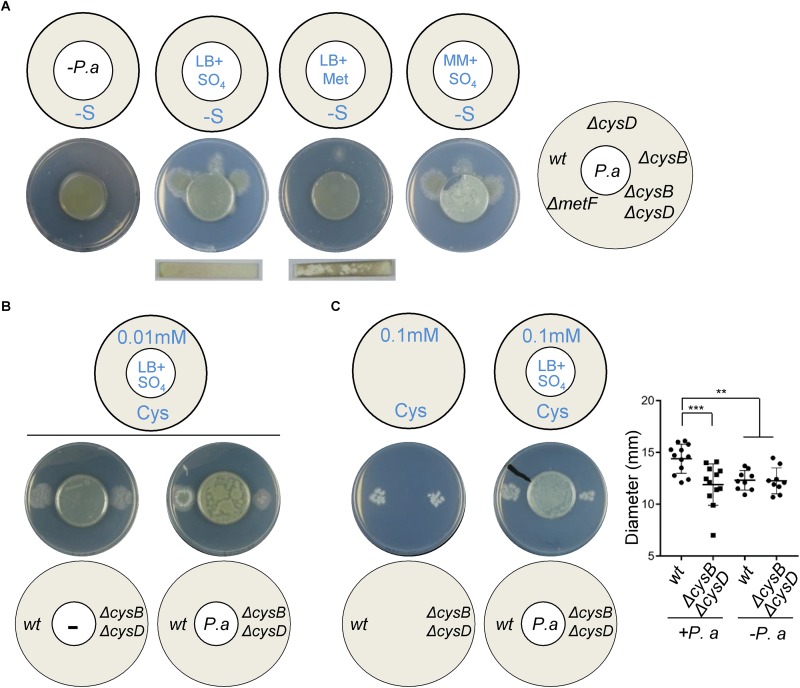
*Pseudomonas aeruginosa* derived VSCs promote *A. fumigatus* growth in sulfur limiting conditions. **(A)** When *P. aeruginosa* (*P.a*) was inoculated on a sulfate containing inner plate it was able to trigger distal growth of *A. fumigatus* wild-type, Δ*cysB*, and Δ*cysD* strains on the outer S-free plates. The Δ*metF* and Δ*cysB*Δ*cysD* strains could not utilize *P. aeruginosa* derived VSCs as a sulfur source. The use of H_2_S strips showed that *P.a* produced a small amount of H_2_S on sulfate and more on methionine, but this could not cross-feed *A. fumigatus*. Plates were incubated at 37°C for 3 days. **(B)** On media containing very low cysteine (0.01 mM) as the sole S-source, both wild-type Δ*cysB*Δ*cysD* strains displayed limited growth. *P. aeruginosa* derived VSCs significantly enhanced *A. fumigatus* wild-type, not mutant, growth. Plates were incubated at 37°C for 3 days. **(C)** Measurement of colony diameters at 36 h post-inoculation on media containing low cysteine (0.1 mM) showed that *P. aeruginosa* derived VSCs significantly stimulated early *A. fumigatus* wild-type, not mutant, growth. Plates were incubated at 37°C for 36 h. ^∗∗^*P* < 0.01 and ^∗∗∗^*P* < 0.001.

It has recently been reported that *P. aeruginosa* promotes *A. fumigatus* distal growth *in vitro* through the production of DMS (Briard et al., 2016). To determine if the exploitation of VSCs as a sulfur source drives this stimulatory effect, we tested the effect of *P. aeruginosa* VSCs on distal growth of *A. fumigatus* wild-type and Δ*cysB*Δ*cysD* strains (Figure 3). *P. aeruginosa* derived VSCs triggered growth of wild-type *Aspergillus*, but not of the Δ*cysB*Δ*cysD* mutant, on S-depleted media (Figure 3A). Actually, the growth profile of the *A. fumigatus* mutants was identical to that observed in Figure 2C, indicating that they are assimilated through the same route. In contrast to *A. fumigatus*, *P. aeruginosa* produced VSCs (i.e., triggered distal fungal growth) when the medium contained an excess of sulfate, but not when it contained an excess of methionine, even if the production of H_2_S was higher from methionine (Figure 3A). This suggests that the bacterium may be producing VSCs through a different mechanism and also supports our previous observation that sulfide cannot be directly exploited as an S-source.

*Aspergillus fumigatus* most likely encounters low concentrations of organic S-compounds to exploit as S-sources during its growth in living tissues (personal observations, reference (Amich et al., 2016) and explained in discussion). Therefore, we tested the capacity of *P. aeruginosa* derived VSCs to enhance fungal growth on low concentrations of cysteine. On very low cysteine (0.01 mM), both *A. fumigatus* wild-type and Δ*cysB*Δ*cysD* strains displayed limited growth; interestingly *P. aeruginosa* derived VSCs significantly enhanced wild-type, but not mutant, growth (Figure 3B). On mid-low cysteine (0.1 mM) both *A. fumigatus* strains developed fully grown colonies at day 3 (Supplementary Figure S3C), but *Pseudomonas* derived volatiles exclusively stimulated early growth (day 1.5) of the wild-type strain (Figure 3C). These results show that *A. fumigatus* can exploit VSCs and cysteine as S-sources simultaneously; therefore the presence of VSCs can enhance fungal growth of the wild-type strain in otherwise limiting conditions.

Contrary to the positive effect of *P. aeruginosa*-derived VSCs on fungal growth, *A. fumigatus* derived VSCs from methionine catabolism did not trigger *P. aeruginosa* growth on an S-depleted plate (Supplementary Figure S4), indicating that *P. aeruginosa* cannot assimilate these volatiles.

### *P. aeruginosa* Produces VSCs Inside Infected *Galleria mellonella*

**FIGURE 4:**
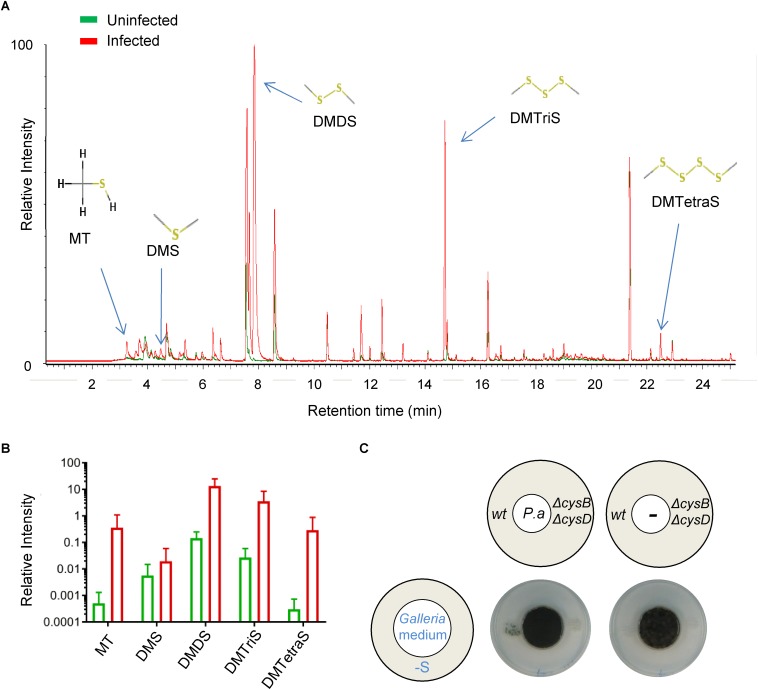
*Pseudomonas aeruginosa* produces VSCs inside infected *Galleria*, which are exploited as S-sources by *A. fumigatus.* Gas emitted from infected or uninfected *Galleria* accumulated in the headspace of flasks and was sampled 24 h after infection and analyzed by GC-MS. **(A)** Chromatogram showing an overlay of the peaks for an infected and an uninfected sample. **(B)** Relative normalized intensity (shown in logarithmic scale) of each of the VSCs peaks detected in two independent experiments with five biological replicates. VSCs accumulated in the flask containing infected *Galleria*. **(C)** *P. aeruginosa* (*P. a*) growing on a solid medium prepared from homogenized *Galleria* triggered distal *A. fumigatus* growth on sulfur depleted medium. Plates were incubated at 37°C for 3 days.

To determine if *P. aeruginosa* produces VSCs when growing inside *Galleria*, we actively sampled the headspace within sealed flasks containing groups of ten infected or uninfected larvae 24 h after infection and measured it using GC-MS (Ahmed et al., 2018). We could detect a clear enrichment of several VSCs specifically in the flasks containing infected larvae, namely MT, DMS, DMDS, and dimethyl trisulfide (DMTriS) (Figures 4A,B). This demonstrates that *P. aeruginosa* does produce VSCs inside living *Galleria* during infection that can potentially be exploited by *A. fumigatus* during co-infection. We next tested if *P. aeruginosa* can trigger distal fungal growth from a homogenized *Galleria* (solidified with agar). Indeed *P. aeruginosa* inoculated on the “Galleria medium,” but not the medium alone, triggered distal *A. fumigatus* growth on S-depleted medium (Figure 4C). This proves that *P. aeruginosa* produces VSCs from the molecules contained in the *Galleria* organism than can then trigger distal fungal growth.

### *P. aeruginosa* Derived VSCs Promote *A. fumigatus* Growth *in vivo*, Which Accounts for an Increase in Mortality

**FIGURE 5:**
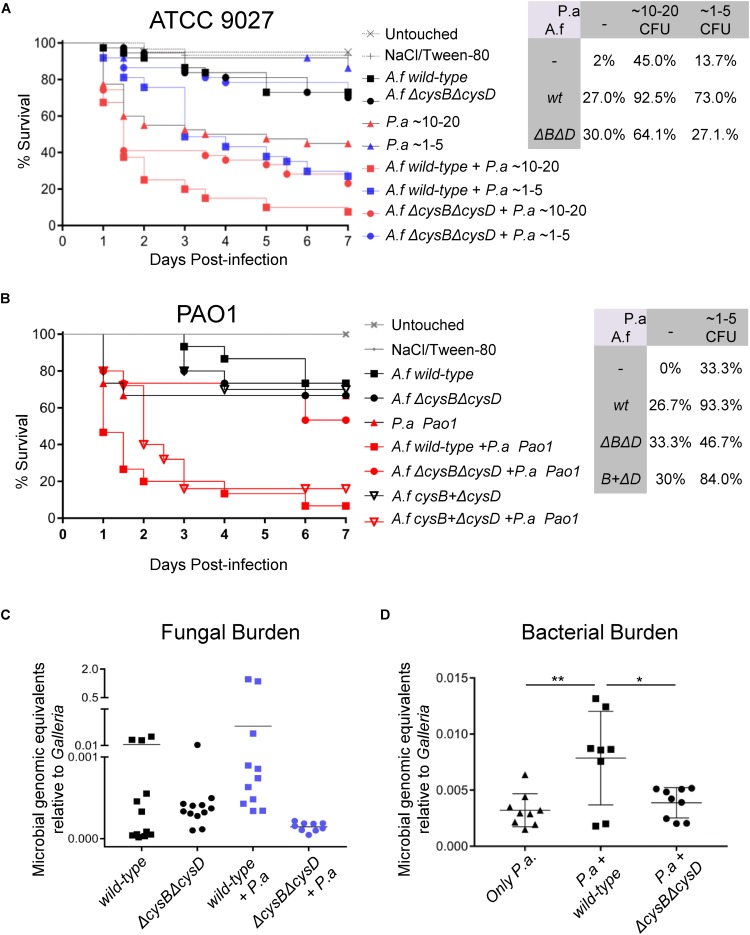
*Pseudomonas aeruginosa* derived VSCs synergize with *A. fumigatus* in co-infection, increasing *G. mellonella* mortality. **(A)** A *G. mellonella* model of infection with a low dose (50 conidia) of *A. fumigatus (A. f)* wild-type or Δ*cysB*Δ*cysD* caused low mortality (27–30%). Infection with a low dose of *P. aeruginosa* ATCC 9027 caused only 13.7% mortality for 1–5 CFU or 45% for 10–20 CFU. *P. aeruginosa* co-infection with *Aspergillus* wild-type dramatically increased mortality to 73% for 1–5 CFUs and to 92.5% for 10–20 CFUs. In contrast, co-infection with the Δ*cysB*Δ*cysD* mutant caused only a moderated increased in mortality to 27.1% for 1–5 CFUs and to 64.1% for 10–20 CFUs (Survival graph shows pool data of 3 independent experiments). **(B)** Co-infection of ∼1–5 CFU *P. aeruginosa* PAO1 with *A. fumigatus (A. f)* wild-type caused a strong increase in mortality (from 26–33% in monomicrobial infections to 93.3%, 2.8 fold). In contrast, co-infection with the *A. fumigatus* Δ*cysB*Δ*cysD* mutant caused a much more moderate increase in mortality (to 46.7%, 1.4 fold). Reintroduction of the *cysB* gene in the Δ*cysB*Δ*cysD* mutant (*cySB* + Δ*cysD*) reconstituted the synergism of mortality of con-infection (84%) (Survival graph shows pool data of 3 independent experiments). **(C)** Four days after infection, *Galleria* co-infected with *A. fumigatus* wild-type and *P. aeruginosa* ATCC 9027 showed a tendency toward having an increased fungal burden and **(D)** had significantly higher bacterial burdens. ^∗∗^*P* < 0.05 and ^∗∗∗^*P* < 0.01.

To elucidate the relevance of VSC for the *in vivo A. fumigatus* – *P. aeruginosa* interaction, we developed a low-dose co-infection model in *Galleria mellonella*. Titration of infectious inocula of the *A. fumigatus* wild-type and Δ*cysB*Δ*cysD* strains determined that 50 conidia caused moderate mortality of 15% (Supplementary Figure S5A). It is notable that Δ*cysB*Δ*cysD* mutant is as virulent as the wild-type (Supplementary Figure S5A), proving that *A. fumigatus* exploits organic S-sources inside the larvae. Titration of the infection inocula for the *P. aeruginosa* clinical isolate ATCC 9027 showed that ∼10–20 CFU caused ∼26.7% mortality and ∼1–5 CFU resulted in ∼100% survival (Supplementary Figure S5B). Co-infection with 50 conidia of *A. fumigatus* wild-type and both ∼1–5 CFU and ∼10–20 CFU of *P. aeruginosa* triggered a ∼45% increase in mortality compared with the single infections (Figure 5A). Thus, relative to monomicrobial infections, co-infection leads to a threefold increase in mortality. Interestingly, co-infection with 50 conidia of Δ*cysB*Δ*cysD* and *Pseudomonas* resulted in no increase in mortality for ∼1–5 CFU and of only 19% for ∼10–20 CFU (Figure 5A), representing a maximum of only a 1.4 fold increase in mortality. Actually, co-infection with *A. fumigatus* wild-type was significantly more virulent than with Δ*cysB*Δ*cysD*: 92.5% vs. 64.1% mortality for ∼10–20 *P. aeruginosa* CFU (*P* = 0.0046) and 73% vs. 27.1% for ∼1–5 *P. aeruginosa* CFU (*P* = 0.001). To corroborate the observed synergism, we also assayed co-infection using the *P. aeruginosa* PAO1 reference strain. PAO1 was similarly virulent as the clinical isolate ATCC 9027 (Supplementary Figure S5C). In agreement with our previous result, co-infection of ∼1–5 CFU *Pseudomonas* PAO1 with *Aspergillus* wild-type synergistically increased mortality (2.8 fold with respect to monomicrobial infections) whilst co-infection with *A. fumigatus*Δ*cysB*Δ*cysD* enhanced mortality to a much lesser extent (1.4 fold) (Figure 5B). Again, co-infection with *Aspergillus* wild-type was significantly more virulent than co-infection with the mutant (93.3% vs. 46.7%, *P* = 0.005). Reintroduction of the CysB encoding gene in the double mutant background (*cysB* + Δ*cysD*) reconstituted the capacity of the strain to cause highest mortality in co-infection with *P. aeruginosa* PAO1 (Figure 5B). These results confirm that co-infection worsens the outcome of infection (i.e., enhances mortality) and suggests that there is a synergistic effect between *P. aeruginosa* and *A. fumigatus* which is significantly dependent on the fungal exploitation of VSCs. To check if this synergy produces enhanced fungal growth, as we had observed *in vitro* (Figure 3B), we measured fungal burden at day 4 post-infection in single and co-infected larvae by qPCR using a published quantification method (Gago et al., 2014; Figure 5C). We observed a general tendency toward increased fungal burden (∼0.25 genome equivalents) in larvae co-infected with *A. fumigatus* wild-type. Taking into account that several co-infected *Galleria* had already succumbed, and so were not included but likely would have had high burdens, we believe this increase is relevant. We also observed a significantly increased bacterial burden in *Galleria* co-infected with *A. fumigatus* wild-type, suggesting a reciprocal synergistic effect of the fungus on *P. aeruginosa* (Figure 5D).

### *P. aeruginosa* Produces VSCs From Artificial Sputum Medium (ASM) and From Mucins

**FIGURE 6:**
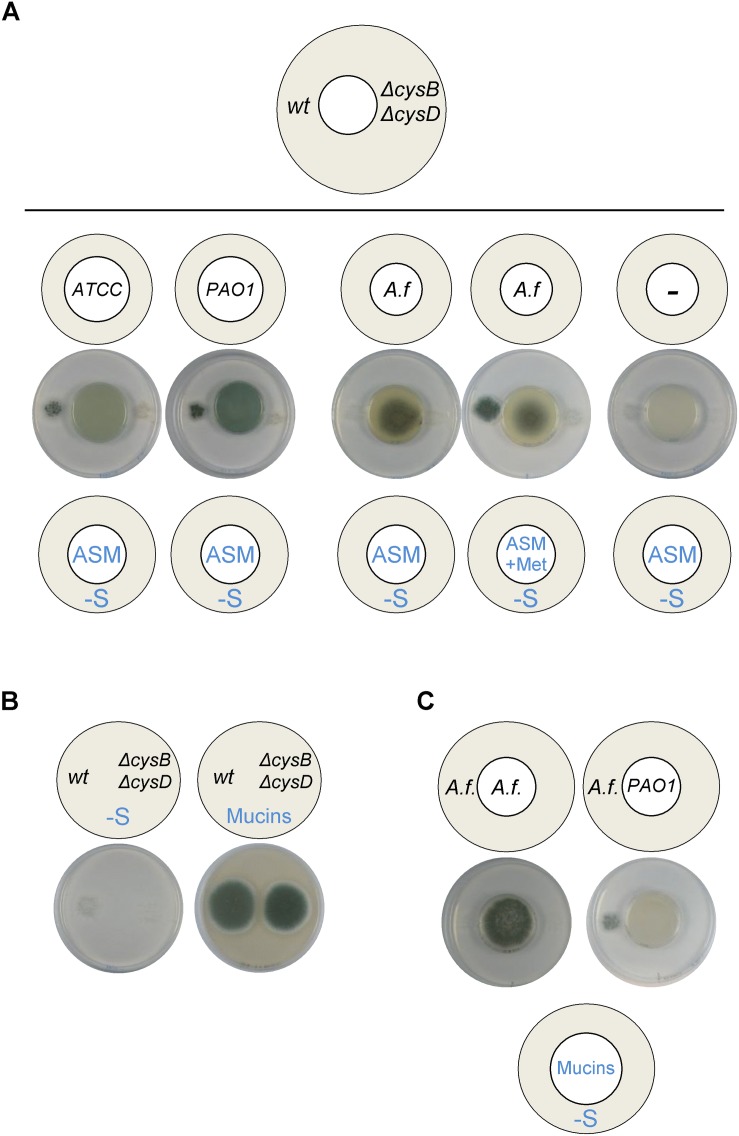
*Pseudomonas aeruginosa* produces VSCs from artificial sputum medium (ASM) and from mucins. **(A)** Growth of *P. aeruginosa* ATCC 9027 and PAO1 strains on ASM triggered distal *A. fumigatus (A. f)* growth. *A. fumigatus* needed supplementation of methionine to the ASM medium to trigger distal growth (i.e., to produce VSCs). **(B)** *A. fumigatus* wild-type and Δ*cysB*Δ*cysD* strains could exploit mucins as the sole sulfur source. **(C)** When grown on mucins as the sole S-source wild-type *P. aeruginosa*, but not *A. fumgiatus*, produced VSCs, which triggered distal *A. fumigatus* growth. Plates were incubated at 37°C for 3 days.

To better mimic the conditions that the pathogens encounter in the airways of CF patients, we used a partially defined medium with a composition that closely resembles CF sputum (Kirchner et al., 2012). Both *P. aeruginosa* wild-type strains ATCC 9027 and PAO1 were able to grow on ASM and produced VSCs that triggered *A. fumigatus* wild-type, but not Δ*cysB*Δ*cysD*, distal growth on S-depleted media (Figure 6A). *A. fumigatus* was able to grow on ASM, but did not produce VSCs unless methionine was supplemented to the medium (Figure 6A). Mucins are cysteine rich proteins and, accordingly, both *A. fumigatus* wild-type and Δ*cysB*Δ*cysD* strains could exploit them as the sole S-source (Figure 6B), but volatiles were not derived (Figure 6C). In contrast, *P. aeruginosa* could produce VSCs from mucins that triggered growth of the distally inoculated *A. fumigatus* wild-type strain. Taken together, these results show that *P. aeruginosa* produces VSCs from mucins contained in ASM that can stimulate *A. fumigatus* growth, suggesting that bacterial utilization of mucins may release an additional exploitable sulfur source for the fungus.

### *P. aeruginosa* Can Cross-Feed *A. fumigatus* When Growing on Murine Lung Tissue and on Human Bronchial Epithelial Cells

**FIGURE 7:**
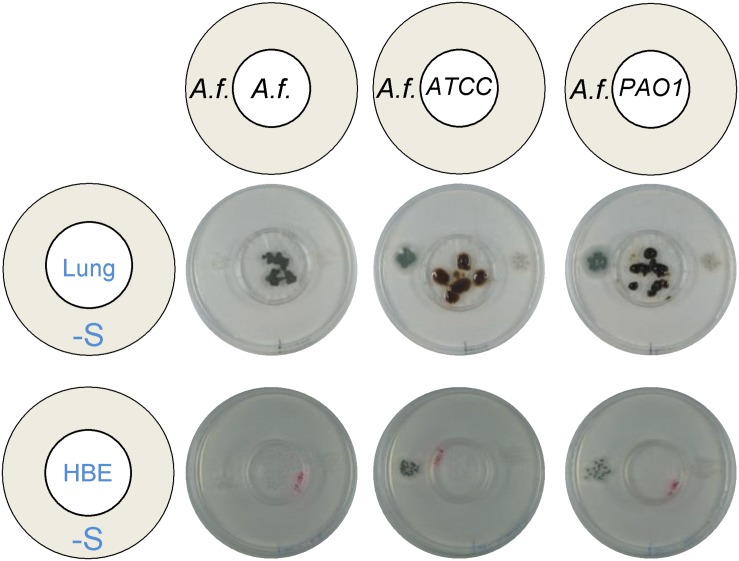
*Pseudomonas aeruginosa* produces VSCs from substrates that replicate the lung. Both *A. fumigatus (A. f)* and *P. aeruginosa* ATCC 9027 and PAO1 strains could grow directly on explanted diced murine lungs and on human bronchial epithelial cells (16-HBE), but only *P. aeruginosa* produced volatiles that triggered distal growth of *A. fumigatus* on a sulfur-free plate. Plates were incubated at 37°C for 3 days.

Aiming to determine if the observed synergistic effect may be relevant for co-infection in humans, we tested the capacity of *P. aeruginosa* to trigger distal *A. fumigatus* growth when inoculated on substrates that replicate the lung. We first inoculated *P. aeruginosa* directly on explanted diced murine lungs and observed that indeed bacterial growth on the lung tissues could trigger growth of distal *A. fumigatus* wild type, but not Δ*cysB*Δ*cysD*, on S-depleted media (Figure 7). *A. fumigatus* was able to grow on diced lungs, but its growth did not cross-feed a distally inoculated colony. We then inoculated *P. aeruginosa* on human bronchial epithelial cells 16HBE and observed that the bacteria is also able to trigger distal *A. fumigatus* wild-type, not Δ*cysB*Δ*cysD*, growth when feeding exclusively from human cells (Figure 7). *A. fumigatus* growth directly on cells was quite limited and did not cross-feed the distal colony. In conclusion, the cross-feeding effect took place when *P. aeruginosa* grew on substrates that mimic the airways, indicating that the observed synergism is likely to take place in human co-infection.

## Discussion

The relevance of pathogen-pathogen interactions in polymicrobial infections is being more and more recognized (Singer, 2010; Peters et al., 2012). However, *A. fumigatus – P. aeruginosa* interactions in co-infection are largely unknown. Several *in vitro* studies have reported negative effects of *P. aeruginosa* on *A. fumigatus* (Kerr et al., 1999; Mowat et al., 2010; Penner et al., 2016; Shirazi et al., 2016; Briard et al., 2017; Sass et al., 2018). However, clinical evidence suggests the opposite, indicating a synergistic association *in vivo*. For instance, a longitudinal study in adolescents with CF found that *A. fumigatus* was the only species associated with an increased risk for infection with *P. aeruginosa* (Hector et al., 2016) and another recent study showed that *P. aeruginosa* infection in CF patients was associated with higher incidence of subsequent *Aspergillus* infection (Granchelli et al., 2018). In addition, antibiotic treatment targeting *Pseudomonas* has been observed to reduce *Aspergillus’* presence in CF patient’s sputum (Baxter et al., 2013b). Most of the negative effects described *in vitro* were observed when setting the pathogens in direct contact. However, given the size of the human lungs, we speculate that the organisms likely occupy physically separated niches, and therefore the action of long distance acting molecules should be very relevant. Surprisingly, despite the increasing number of studies investigating pathogen-pathogen interactions, the role of volatiles in intermicrobial cross-talk *in vivo* has been largely overlooked. Recently it has been described that the *Pseudomonas* derived volatile sulfur compound DMS promotes *A. fumigatus* growth *in vitro* (Briard et al., 2016). In this study we have scrutinized the pathway for VSCs assimilation in *A. fumigatus* and explored the hypothesis that *Pseudomonas* derived VSCs could be utilized by *A. fumigatus in vivo*, producing a synergistic effect in co-infection.

We have shown that *A. fumigatus* assimilates VSCs through the action of CysB (cysteine synthase) or CysD (homocysteine synthase). The double mutant Δ*cysB*Δ*cysD* cannot feed from inorganic sources (nor incorporate VSCs), but is completely virulent in both the *G. mellonella* and leukopenic murine models of infection (Supplementary Figures S5A, S6A). This strongly suggests that *A. fumigatus* predominately feeds from organic S-sources within the tissues of both the larvae and the mammalian lung. We have shown that in the *Galleria* model, co-infection of *P. aeruginosa* with wild-type *A. fumigatus* enhances virulence to a greater extent than co-infection with the Δ*cysB*Δ*cysD* strain. It has been recently described that co-infection triggers a more pronounced pro-inflammatory response (Bergeron et al., 2017); indeed Bergeron et al. (2017) found a stronger immune response in *Candida albicans* – *P. aeruginosa* co-infection, which they proposed could be the reason of the observed increment in virulence and mortality. Therefore, it could be argued that in our model a stronger inflammatory response accounts for the increased mortality of co-infection, and that the double mutant is more sensitive to such hyperinflammation. However, a conidiocidal assay using human neutrophilic granulocytes showed that neutrophils kill conidia of the wild-type and double mutant strains similarly (Supplementary Figure S6B), indicating that the Δ*cysB*Δ*cysD* strain is not more susceptible to attack by immune effector cells. Therefore, although a stronger pro-inflammatory response is likely to play a role in the pathology of co-infection, it does not explain the differences found between the *A. fumigatus* wild-type and mutant strains. We have shown that *P. aeruginosa* produces VSCs *in vivo*, which translates into an increased fungal burden in *G. mellonella* larvae co-infected with *A. fumigatus* wild-type, but not Δ*cysB*Δ*cysD*. Interestingly, we have also observed an enhanced bacterial burden in larvae co-infected with wild-type, but not the mutant strain, *Aspergillus*. It has been reported that *A. fumigatus* enhances *P. aeruginosa* production of the virulence factor elastase (Smith et al., 2015). Similarly, it has been shown that *C. albicans* triggers production of *P. aeruginosa* virulence factors, which was suggested, is the leading cause of mortality in co-infection (Trejo-Hernandez et al., 2014). Consequently, we hypothesize that *Pseudomonas* derived VSCs enhance fungal growth, which then somehow stimulates bacterial growth and pathogenesis, contributing to an increase in the mortality of co-infection. Very recently Reece et al. (2018) have reported that *P. aeruginosa* causes increased mortality in *G. mellonella* when infected 24 h after a challenge with a non-lethal *A. fumigatus* dose. The authors proposed that this effect is likely due to one of two options: either the fungus is producing immunoregulatory toxins that suppress the immune system of *G. mellonella*, making them more susceptible to *P. aeruginosa* infection or the two species interaction causes a more virulent infection. Even if we believe they are not mutually exclusive and toxins may also play a role, our results support the second hypothesis: inoculated *P. aeruginosa* produces VSCs, which increases *A. fumigatus* growth, and the presence of the fungus enhances bacterial pathogenicity, all resulting in higher mortality. Interestingly, the authors assayed various *A. fumigatus* and *P. aeruginosa* isolates (reference strains and patient isolates) and observed different effects depending on the strain combination of co-infection. Although we have not made any attempt to address this, we speculate it might be partially explained by subtle differences in the volatile production capacities of the *P. aeruginosa* isolates, and/or their responses to interactions that occur *in vivo*.

We have observed *in vitro* that *P. aeruginosa* produces VSCs in the presence of sulfate. It has been shown that *P. aeruginosa* can feed from sulfate present in human respiratory tracts, and that it can also use sulfate contained within mucins as an S-source (Robinson et al., 2012). We have shown that it produces VSCs when grown on Artificial Sputum Medium and on mucins. *G. mellonella* hemolymph contains mucin-like proteins (Li et al., 2002), which we speculate the bacteria is exploiting as an S-source, which triggers VSCs production inside the infected larvae. It had previously been described that *A. fumigatus* is able to degrade mucins (Leger and Screen, 2000), and we have observed that the fungus can use them as a sulfur source *in vitro*. This is most likely due to the utilization of the enriched amino acid cysteine as the S-source, as suggested by the capacity of the Δ*cysB*Δ*cysD* mutant to grow on mucins and the inability of the wild-type to produce VSCs. However, the gene(s) required for utilization of the sulfate contained within the mucins (which could be one or several of the three arylsulphatases encoded by its genome: AFUA_3G02290, AFUA_5G12940, and AFUA_8G02520) may not be expressed in the presence of cysteine (Amich et al., 2013), impeding its exploitation as an additional S-source. Therefore, we propose that during co-infection *P. aeruginosa* liberates an otherwise non-accessible sulfur source in the form of VSCs, which in turns makes the tissues less sulfur limiting for the fungus and promotes its growth. An alternative explanation could be that *P. aeruginosa* liberates and/or releases sulfate or other inorganic sulfur sources, which could be assimilated by *A. fumigatus* wild-type but not Δ*cysB*Δ*cysD*. Although we cannot reject this possibility, we believe it is unlikely because the growth promoting effect would be restricted to the proximity of bacterial cells and also because the genes of the sulfate reduction assimilation pathway (required to assimilate inorganic S-sources) are downregulated in the presence of organic S-compounds (Amich et al., 2013).

Interestingly, we show that when *P. aeruginosa* grows on substrates that resemble the lung, namely explanted murine lungs and human bronchial epithelial cells, it can trigger distal *A. fumigatus* growth. A recent analysis of bronchoalveolar lavage fluid of CF patients detected sulfur-containing volatiles specifically in *P. aeruginosa* infected patients (Nasir et al., 2018). Therefore, it seems plausible that *P. aeruginosa* derived VSCs are able to trigger the synergistic effect described in this manuscript and be relevant for co-infection in the human airways.

In conclusion, we report a volatile dependent synergistic effect of *Pseudomonas aeruginosa* on *A. fumigatus* during co-infection of *G. mellonella* that causes higher fungal and bacterial burdens, resulting in increased mortality. This effect is likely relevant for the pathogen-pathogen interaction in patients’ lungs and thus has an impact on the outcome of co-infection.

## Materials and Methods

### Strains, Media and Culture Conditions

The *Escherichia coli* strain DH5α (Woodcock et al., 1989) was used for cloning procedures. Plasmid-carrying *E. coli* strains were grown at 37°C in LB liquid medium under selective conditions (100 μg⋅mL^–1^ ampicillin, 50 μg⋅mL^–1^ kanamycin or 10 μg⋅mL^–1^ gentamycin); Media was solidified with 1.5% agar. All plasmid constructs used in the course of this study are listed in Supplementary Table S1 and were generated using the Seamless Cloning (Invitrogen) technology as previously described (Amich et al., 2013, 2016).

The wild-type clinical isolate *A. fumigatus* strain ATCC 46645 served as reference recipient; gene knock-out derivative strains of this isolate were generated using a standard protoplasting protocol (Szewczyk et al., 2006). Once verified by Southern-blot, the mutants were grown on xylose to activate the encoded beta-recombinase, which self-excise the selection marker, a strategy previously described (Amich et al., 2013, 2016) (Supplementary Figure S7). Reversion of the *cysB* gene was directed to its natural locus using homology flanking regions. Mutants were selected by reconstitution of ability to grow on sulfate as sole sulfur source and checked by PCR. *A. fumigatus* strains were generally cultured on minimal medium (MM) (Amich et al., 2016) at 37°C. For selection in the presence of resistance markers 50 μg⋅mL^–1^ of hygromycin B or 100 μg⋅mL^–1^ of pyrithiamine (InvivoGen) was applied. In sulfur-free medium, MgCl_2_ substituted for MgSO_4_, and a modified mixture of trace elements lacking any sulfate salt was used. Artificial sputum medium (ASM) was prepared as previously described (Kirchner et al., 2012) and solidified with 1.5% agar.

*Pseudomonas aeruginosa* wild-type clinical isolate ATCC 9027 and reference strain PAO1 were used for cross-feeding experiments and *G. mellonella* infections. Strains were routinely grown on LB, which was supplemented with 5 mM methionine or 2 mM Na_2_SO_4_ for some experiments. “Galleria medium” was prepared by homogenizing a larva with sterile ultrapure water on a 70 μm cell-strainer (Falcon) with the use of a syringe plunger. This homogenate was mixed with agar (to 1.5% final concentration), solidified, and illuminated with UV light for 10 min to reduce bacterial contamination. Murine lungs were explanted from uninfected controls at the end of a different experiment. 16HBE cells (Cozens et al., 1994) were seeded in coated plates (Nunclon^TM^ Delta, ThermoFisher Scientific) with Dulbecco’s Modified Eagle Medium (DMEM, Gibco^®^) at a very high density (>100% confluency), left overnight to adhere, and then the medium was removed to inoculate *P. aeruginosa* directly on the cells. For *G. mellonella* infection, one single colony of an overnight grown plate was suspended in 1 mL NaCl 0.9%/Tween-20 0.002%. This solution was diluted serially to yield the desired concentrations, as confirmed each time by plating the dilutions on LB.

For cross-feeding experiments one colony of *P. aeruginosa* or 10^3^ conidia of *A. fumigatus* were inoculated in the internal plate, and 10^2^ conidia of each *A. fumigatus* strain in the external petri dish. Unless otherwise stated, plates were incubated at 37°C for 3 days inside zip-closed plastic bags. Na_2_S, DMS, and DMDS were purchased from Sigma and the amounts described in each experiment were added directly to Whatman filter paper placed inside the inner plate.

### Extraction and Manipulation of Nucleic Acids

Standard protocols of recombinant DNA technology were carried out (Sambrook et al., 1989). Phusion^®^ high-fidelity DNA polymerase (Thermo Fisher Scientific) was generally used in polymerase chain reactions and essential cloning steps were verified by sequencing. Fungal genomic DNA was prepared following the protocol of Kolar et al. (1988) and Southern analyses were carried out as described (Southern, 1975, 2006), using the Amersham ECL Direct Labeling and Detection System^®^ (GE Healthcare).

### Detection of Volatile Sulfur Compounds Derived From *Aspergillus fumigatus in vitro*

Volatile sulfur compounds measurement was performed as described (Briard et al., 2016). Briefly, 10^6^ conidia per ml *A. fumigatus* spores were inoculated in 5 mL of sulfur free MM with 2 mM Na_2_SO_4_ or 5 mM methionine as sulfur source and incubated in 40 mL vials with screw caps with a rubber/PTFE septum for 3 days at 37°C. Direct sampling was performed in the headspace of culture vials using SPME fibers [2 cm composite coating carboxen/divinylbenzene/polydimethylsiloxane (Car/DVB/PDMS)]. Vial septa were pierced with the needle of the filament holder followed by fiber exposure for 30 min in the incubator at 37°C. Volatiles were injected into Agilent HP-5MS GC-MS column (length 30 m, internal diameter 0.25 mm, and film thickness 0.25 μm) using a Front SS Inlet He in Pulsed Splitless mode.

### MT Oxidase Activity

*Aspergillus fumigatus* protein extracts were isolated from ground mycelia in lysis buffer (50 mM HEPES; 1% NP-40; 0.5 mM EDTA; 0.1% SDS; 1mM DTT) with 1× complete protease inhibitor cocktail (Roche). 500 μM methanethiol was added or not to 0.5 mg protein extracts in 200 μl of lysis buffer and incubated for 6 h at 37°C in sealed tubes. Formaldehyde was measured using Purplad^®^ reagent (Sigma) as previously described (Boden et al., 2011). Briefly, 100 μl of a 34 mM dilution of Purpald^®^ in 2 M NaOH was added to the tubes, incubated for 15 min and OD measured at 550 nm. The increment in absorbance was normalized with lysis buffer and converted to μM of formaldehyde using a standard curve.

### *Galleria mellonella* Infection

Sixth-stage instar larval *G. mellonella* moths (15 to 25 mm in length) were ordered from the Live Foods Company (Sheffield, United Kingdom). Infections were performed according to Kavanagh and Fallon (2010). Randomly selected groups of 15 larvae were injected with 20 μl of NaCl 0.9%/Tween-20 0.002% containing varying doses of *A. fumigatus* conidia or *P. aeruginosa* CFUs, as detailed in each experiment. *A. fumigatus* was always injected into the last right proleg, whilst *P. aeruginosa* was always injected in the left proleg. Braun Omnican 50-U 100 0.5-mL insulin syringes with integrated needles were used. In each experiment an untouched and a saline injected control were included, to verify that mortality was not due to the health status of the larvae or the injection method.

### Detection of Volatile Sulfur Compounds Derived From *Pseudomonas aeruginosa* in Infected *Galleria*

The headspace of flasks containing 10 *G. mellonella* uninfected or infected with *P. aeruginosa* (5 flasks/group) were sampled after a 24 h incubation at 37°C, using a method described previously (Ahmed et al., 2018). Briefly, a total volume of 200 mL was sampled from a 100 mL glass flask containing *Galleria*, at a flow of 100 mL/min, onto conditioned silco-treated stainless steel multi-sorbent tubes packed TenaxTA/Carbograph5TD. This sorbent bed was chosen to maximize the range of Volatile Organic Compounds (VOCs) captured, whilst minimizing water retention. Sorbent tubes were stored at 4°C before analysis, for up to 7 days.

Volatile organic compounds trapped onto the sorbent bed were analyzed using thermal desorption-gas chromatography-mass spectrometry (TD-GC-MS). In summary, sorbent tubes underwent a two-stage thermal desorption process where the initial desorption was carried out at 220°C for 5 min. Prior to this, an internal standard (1 ppmV 4-bromoflorobenzene in N_2_) was injected onto each tube. VOCs were purged off the tube using N_2_ and onto a cold trap (kept at 0°C), which was subsequently desorbed at 230°C for 3 min. VOCs were then transferred to the GC column at a constant flow of 2 mL/min using He as the carrier gas. The Agilent DB-1ms GC column (length 60 m, internal diameter 0.25 mm, and film thickness 0.5 μm) was housed within an Agilent 6890 GC oven (Agilent Technologies, West Lothian, United Kingdom) which underwent a ramped programs with an initial 40°C temperature (no hold), ramped to 250°C (18 min hold) at 5°C/min. The total GC cycle time was 60 min. After separation by GC, VOCs were transferred to a Waters GCT mass spectrometer (Waters Corporation, Manchester, United Kingdom), equipped with an EI + source (70 eV), and a *time-of-flight* mass analyzer. Data were acquired between *m/z* 29–400 in centroid mode, at a rate of 10 scans/s.

Data were analyzed by using Target Lynx (Masslynx software version 4.1, Waters Corporation, Manchester, United Kingdom). The exact masses of base peaks *m/z* 47, 62, 94, 126, and 158 were used to extract methanethiol, dimethyl sulfide, dimethyl disulfide, DMTriS, and dimethyl tetrasulfide, respectively, along with retention indices calculated using alkane chemical standards (C_5_–C_15_). Data were pre-processed using the Apex Trak algorithm (Waters Corporation, Manchester, United Kingdom) which included peak integration, smoothing and baseline correction. Data were then normalized to the internal standard (with an extracted base peak of *m/z* 174). DMS and DMDS were identified to MSI level 1 standard (Fiehn et al., 2007), a NIST library search was performed on the sample peak, together with retention time matching of their counterpart external standards. Other VSCs were identified to MSI level 2 i.e., NIST library search only.

### Neutrophil Killing Assay

Human neutrophils were isolated from healthy donors’ blood using the EasySep^TM^ Direct Human Neutrophil Isolation Kit (StemCell) following the manufacturer’s instructions for Easystep^TM^ Magnets EasyEights^TM^ protocol. Purified neutrophils were set in culture in RPMI medium (Sigma) supplemented with 10% human serum (isolated from the same donor’s blood by centrifugation at 1200 × g 4 min), and conidia were added on top at a multiplicity of infection (MOI) ratio of 1:10. After 6 h, medium was collected and centrifuged at 4,000 rpm for 5 min; the pellet was resuspended in distilled water and finally resuspended in 1 ml 0.9% NaCl plus 0.01% Tween 20. Different dilutions were plated onto MM + cysteine in triplicates, and colony forming units were counted. For input controls, the same amount of conidia were incubated in RPMI + serum for 6 h without neutrophils and treated and plated as the experimental conditions.

### Leukopenic Murine Model of Pulmonary Aspergillosis

The experiment was performed under United Kingdom Home Office project license PPL70/7324 and approved by the University of Manchester Ethics Committee. Outbred CD1 male mice (22–26 g) were purchased from Charles Rivers and left to rest for at least 1 week before the experiment. Mice were allowed access *ad libitum* to water and food throughout the experiment. Mice were immunosuppressed with 150 mg/kg of cyclophosphamide on days −3 and −1 and with 112 mg/kg cortisone acetate on day −1. On day 0 mice were anesthetized with isofluorane and intranasally infected with a dose of 10^5^ conidia (40 μl of a freshly harvested spore solution of 2.5 × 10^6^ conidia/mL). Immunosuppression was maintained by injecting 150 mg/kg cyclophosphamide every 3 days (days + 2 and + 5) and survival was followed for 7 days.

### Statistical Analyses

All graphs were prepared using GraphPad Prism v7.04 software. For statistical analysis of survival curves the Log-Rank test was applied. In column graphs one-way ANOVA or Kruskal-Wallis (depending on the distribution) were utilized to determine differences among groups. Significance was considered when ^∗^*P* < 0.05, ^∗∗^*P* < 0.01, and ^∗∗∗^*P* < 0.001.

## Data Availability Statement

Datasets for GC/MS experiments are available upon reasonable request to the corresponding author. All other datasets are included in the manuscript/Supplementary Files.

## Ethics Statement

The animal study was reviewed and approved by the UK Home Office project license PPL70/7324 and approved by the University of Manchester Ethics Committee.

## Author Contributions

JS performed the majority of the experiments and wrote the manuscript. MS-O helped JS with most of the experiments. WA performed and analyzed the *in vivo* measurements of VSCs. CH performed and analyzed the *in vitro* measurements of VSCs. CZ helped with the acquisition and analysis of volatile measurements. RT provided the support with *Galleria* experiments. MB supported the design of *in vivo* volatile experiments and revised the manuscript. J-PL supported the design *in vitro* volatile experiments and revised the manuscript. SK participated in the design of cross-feeding experiments and revised the manuscript. SF devised the volatile measurements and revised the manuscript. EB participated in the conception of the study and contributed to manuscript writing. JA designed the project and wrote the manuscript.

## Conflict of Interest

The authors declare that the research was conducted in the absence of any commercial or financial relationships that could be construed as a potential conflict of interest.
