# Supplementary material for: Pseudomonas aeruginosa-Derived Volatile Sulfur Compounds Promote Distal Aspergillus fumigatus Growth and a Synergistic Pathogen-Pathogen Interaction That Increases Pathogenicity in Co-infection
Source: Front Microbiol. 2019 Oct 9;10:2311. doi: 10.3389/fmicb.2019.02311 (PMC6794476; doi:10.3389/fmicb.2019.02311)
Supplement: Supplementary file 1 [file Data_Sheet_1.pdf]

Fig. S1

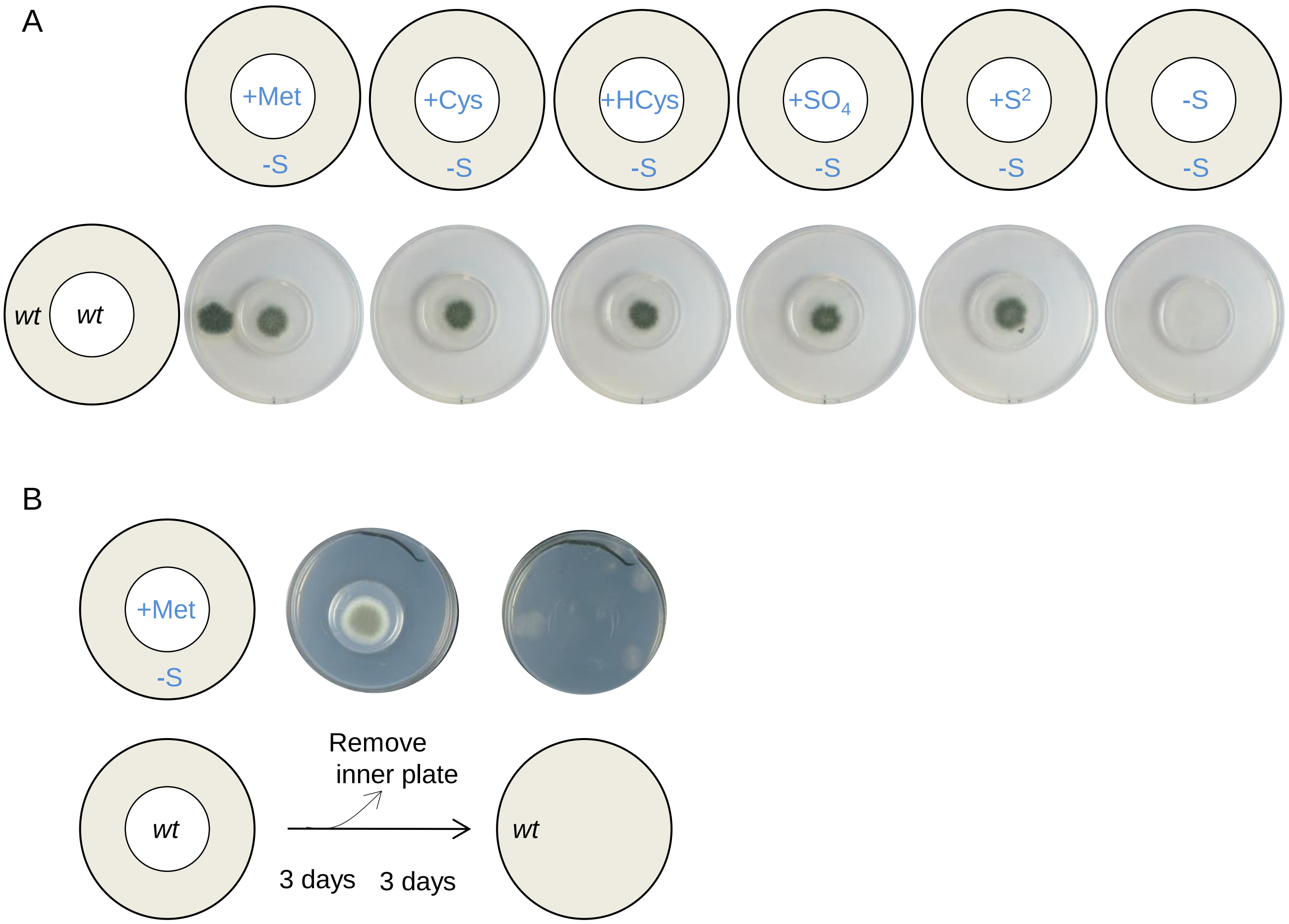

Fig S1. *Aspergillus fumigatus* assimilates VSCs derived from the catabolism of methionine seemingly from the air.

A) *A. fumigatus* was inoculated on internal plates containing various different sulfur sources. Only growth on methionine triggered distal growth on the sulfur free outer plate. Plates were incubated at 37 °C for 3 days. B) *A. fumigatus* was inoculated in the inner methionine containing plate and incubated for 3 days. After that time, the inner plate was removed, the outer plate ventilated and *A. fumigatus* inoculated on the outer S-free plate. The fungus could not grow on the outer plate..

Fig. S2

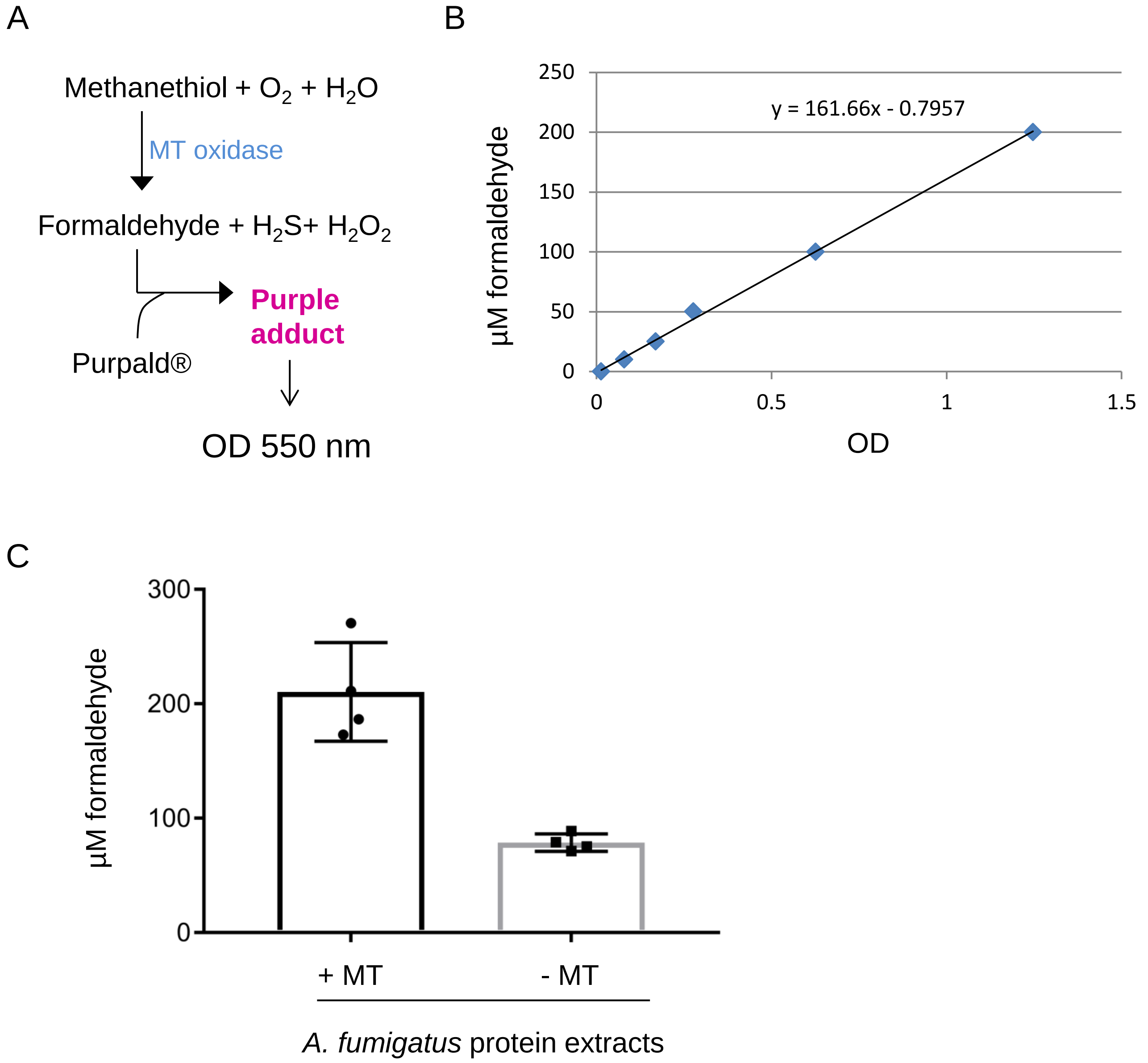

Fig S2. Methanethiol oxidase activity assay.
A) Outline of the colorimetric detection method employed. B) Stand curve of formaldehyde diluted in lysis buffer (see material and methods). C) Concentration of formaldehyde detected in *A. fumigatus* protein extracts with and without addition of methanethiol (MT).

Fig. S3

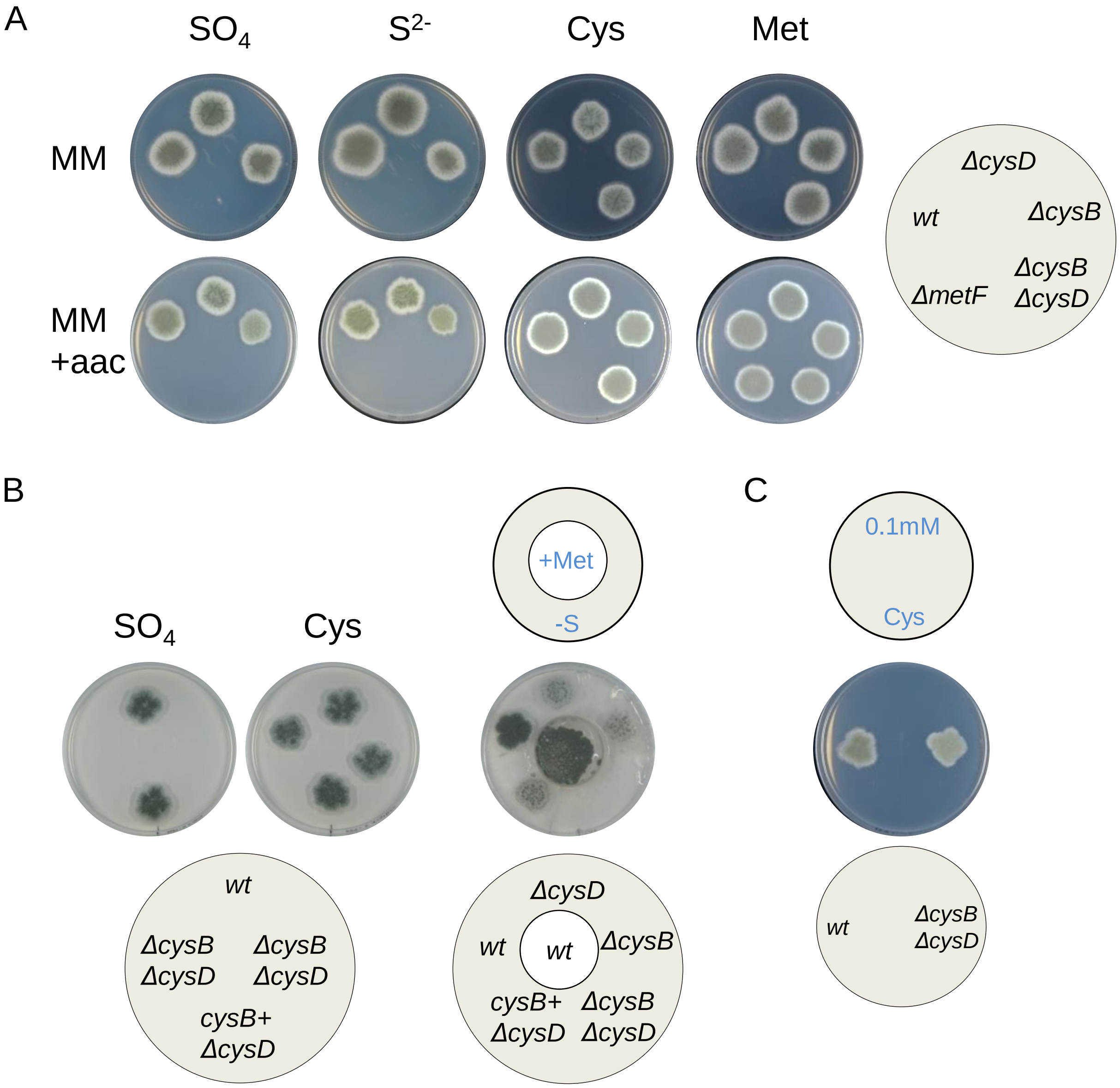

Fig S3. Phenotypic study of *A. fumigatus* mutants on different S-sources.
A) The *ΔcysBΔcysD* double mutant could not grow on inorganic S-sources, but grew on organic sources. The *ΔmetF* strain needed methionine supplementation, as it is a methionine auxotroph since it cannot recycle the methionine synthase co-substrate 5, methyl-THF. The media had to be further supplemented with a mix of all amino acids (except cysteine and methionine), at 1 mM, to yield *ΔmetF* growth. B) Reintroduction of the *cysB* gene in its natural locus in the *ΔcysBΔcysD* mutant reconstituted the capacity of the strain to grow on sulfate and to cross-feed from *A. fumigatus* derived volatiles. C) Both wild-type and *ΔcysBΔcysD* strains were able to developed fully grown colonies on media containing 0.1 mM cysteine as the S-source 3 days after inoculation. Plates were incubated at 37 °C for 3 days.

Fig. S4

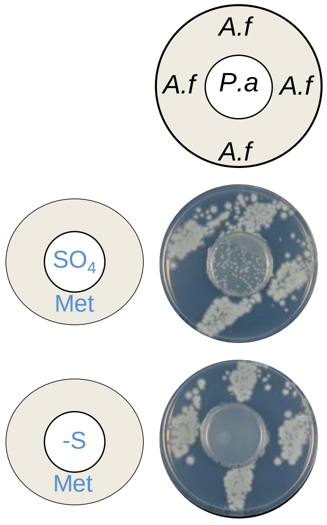

Fig S4. *A. fumigatus* derived VSCs cannot trigger *P. aeruginosa* growth on sulfur-depleted media.

A. fumigatus (*A.f*) VSCs derived from methionine catabolism did not trigger growth of *P. aeruginosa* (*P.a*) on an S-free medium. Plates were incubated for 3 days at 37 °C.

Fig. S5

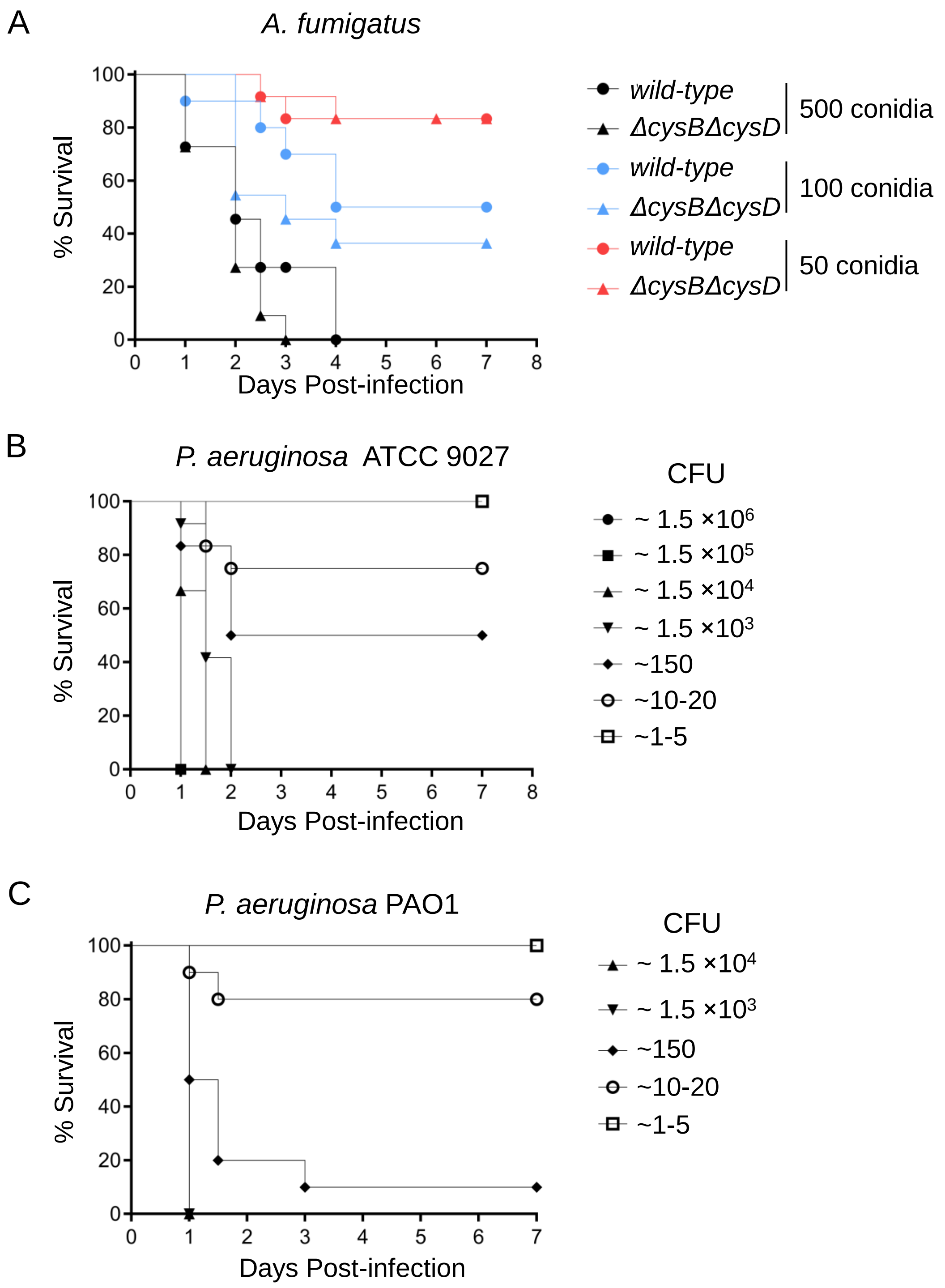

Fig S5. Titration of infection inocula for *A. fumigatus* and *P. aeruginosa*.
A) Titration of *A. fumigatus* wild type and *ΔcysBΔcysD* infection inocula in *Galleria mellonella*. Both strains are equally virulent. A dose of 50 conidia caused only 13.4% mortality. B) Titration of *P. aeruginosa* ATCC 9027 infection inocula in *Galleria mellonella*. A dose of ~10-20 CFU caused 26.7% mortality and a dose of ~1-5 CFU resulted in 100% survival. C) Titration of *P. aeruginosa* PAO1 infection inocula in *Galleria mellonella.* A dose of ~10-20 CFU caused 20% mortality and a dose of ~1-5 CFU resulted in 100% survival.

Fig. S6

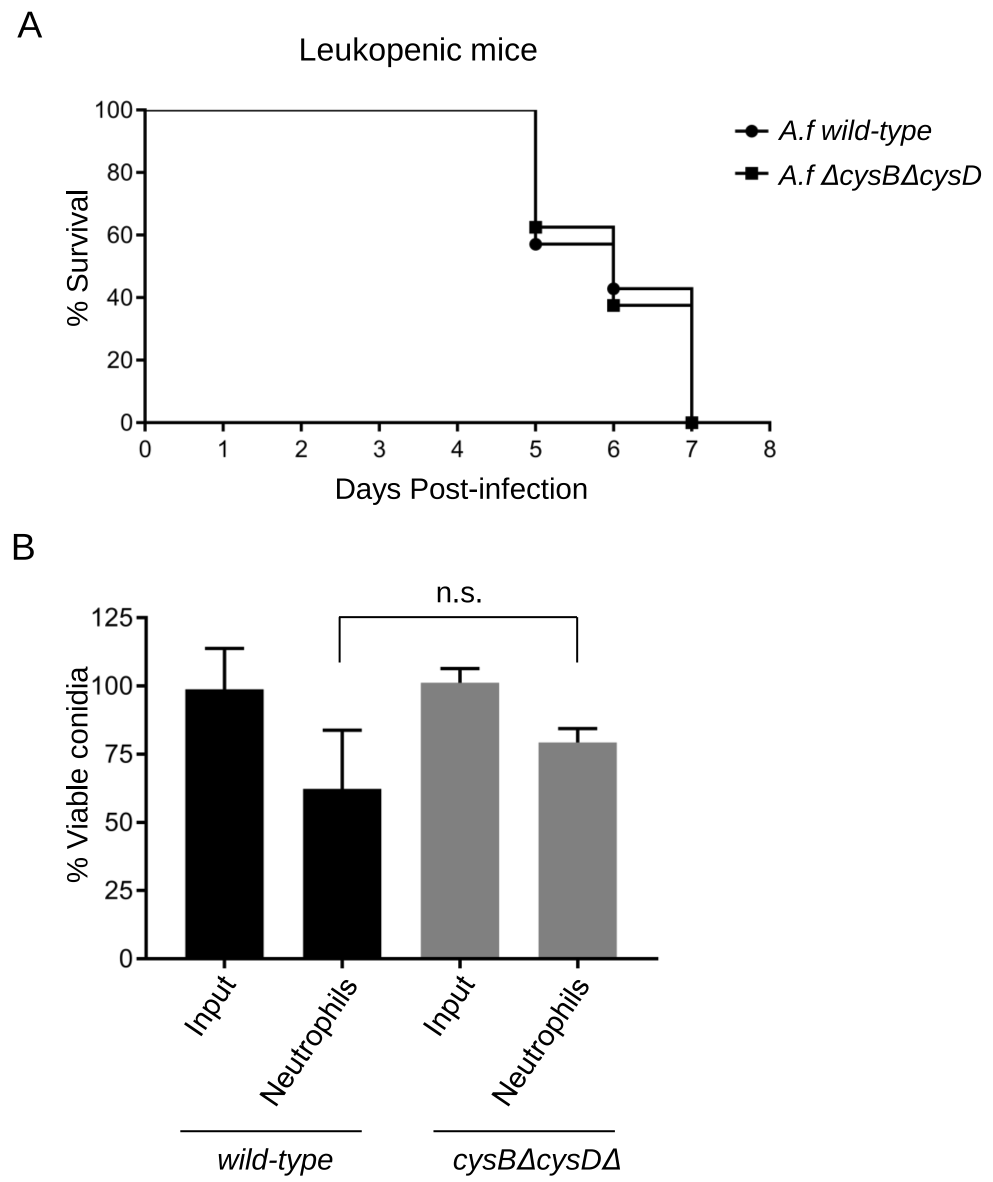

Fig S6. Virulence and neutrophil killing of the *ΔcysBΔcysD A. fumigatus* mutant.

A) In a leukopenic model of pulmonary aspergillosis the *A. fumigatus* wild-type and *cysBΔcysDΔ* strains showed the same virulence, suggesting that *A. fumigatus* feeds from organic S-sources in the murine lung. B) Conidiocidal assay using human neutrophils purified from blood. *A. fumigatus* wild-type and *ΔcysBΔcysD* conidia are killed at similar levels.

Fig. S7

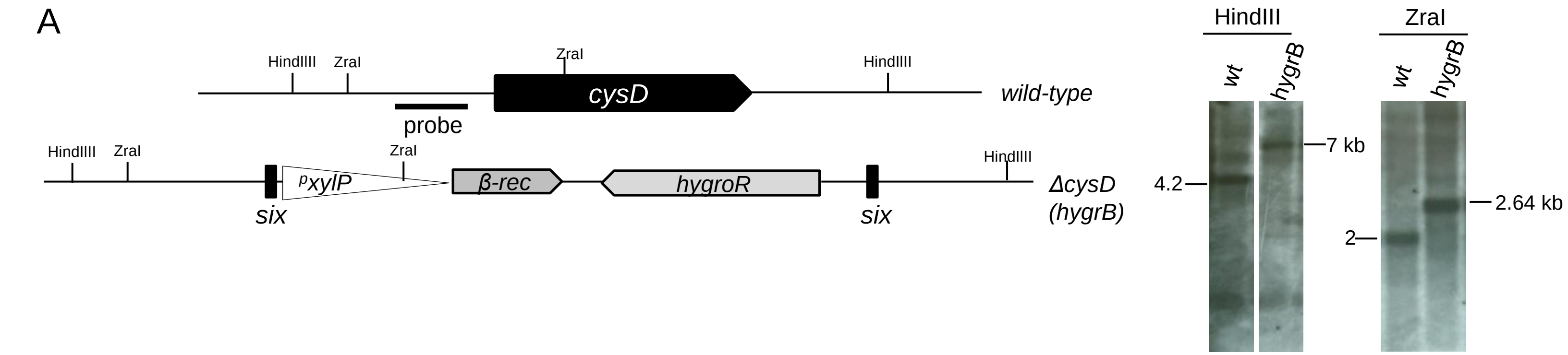

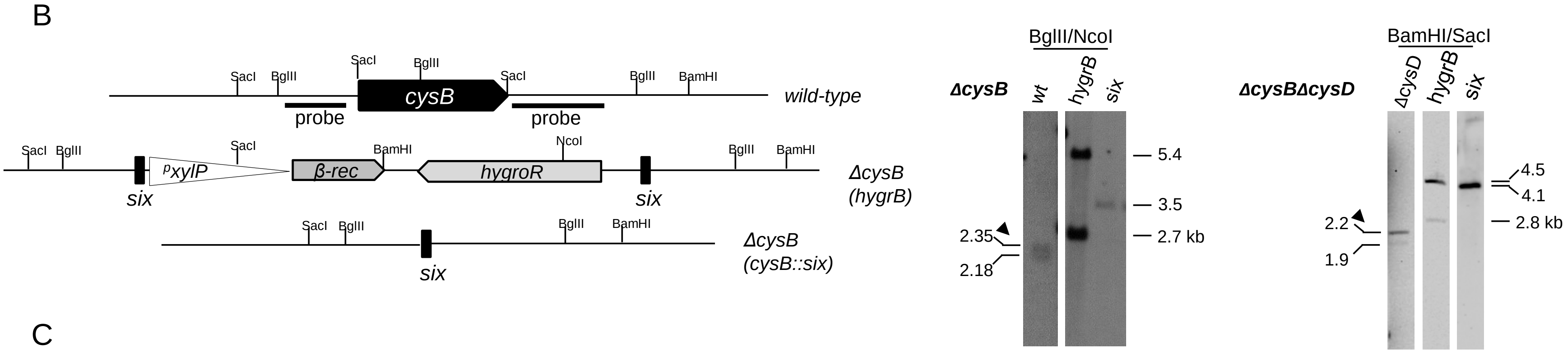

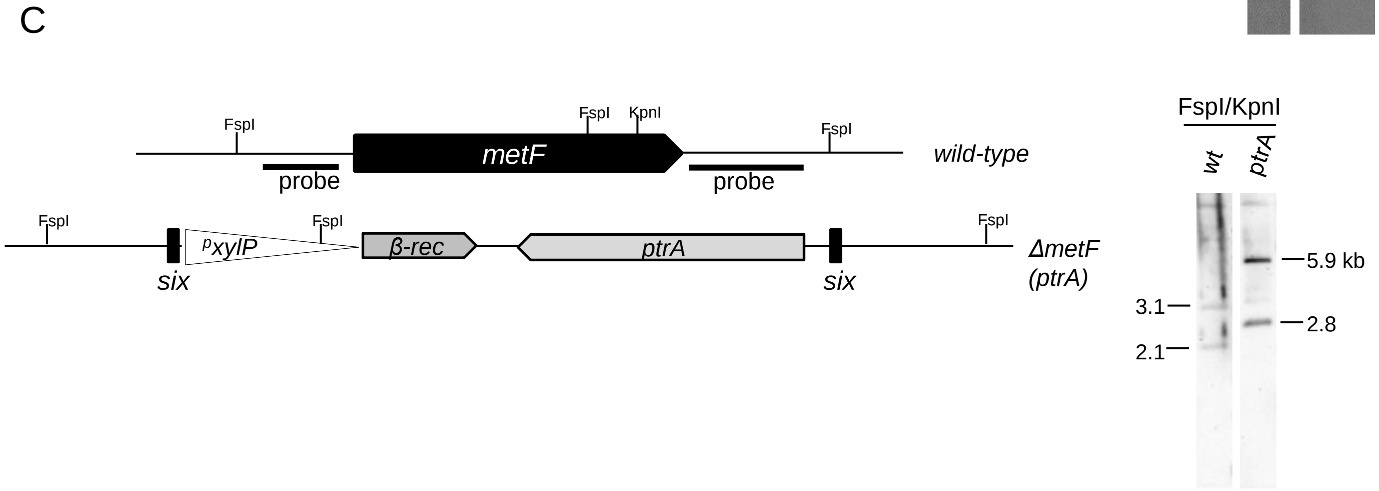

Fig. S7. Southern blots of mutants A) Strategy and blot to confirm *ΔcysD*. B) Strategy and blot to confirm *ΔcysB* in both the wild-type background (single mutant, publish in Amich et al 2016) and *ΔcysD* background (double mutant). C) Strategy and blot to confirm *ΔmetF.*
