# Supplementary material for: Pseudomonas aeruginosa-Derived Volatile Sulfur Compounds Promote Distal Aspergillus fumigatus Growth and a Synergistic Pathogen-Pathogen Interaction That Increases Pathogenicity in Co-infection
Source: Front Microbiol. 2019 Oct 9;10:2311. doi: 10.3389/fmicb.2019.02311 (PMC6794476; doi:10.3389/fmicb.2019.02311)
Supplement: Supplementary file 2 [file Table_1.DOC]

| Plasmid | Description | Reference |
| --- | --- | --- |
| pUC19L | General cloning vector | Invitrogen |
| pSK529 | Carries the β-recombinase gene under a xylose-inducible promoter control and the hygromycin B resistance cassette flanked by *six* attachment sites | Jiménez-Ortigosa *et al* 2012  Amich *et al* 2013 |
| pSK530 | Carries the β-recombinase gene under a xylose-inducible promoter control and the pyrithiamine resistance cassette flanked by *six* attachment sites | This study |
| pJA17 | *A.f. cysB* replacement cassette: β-rec/*six* blaster module from pSK529 flanked by 5´and 3´ *cysB* homology regions | Amich *et al* 2016 |
| pJA27 | *A.f. cysD* replacement cassette: β-rec/*six* blaster module from pSK529 flanked by 5´and 3´ *cysD* homology regions | This study |
| pJA46 | *A.f. metF* replacement cassette: β-rec/*six* blaster module from pSK530 flanked by 5´and 3´ *metF* homology regions | This study |
| pJA89 | *A.f. cysB* ORF flanked by its natural 5´and 3´ regions | This study |

**Table S1**

*Plasmids used in the course of this study*
